# Supplementary material for: Ingestion of Nitrate and Nitrite and Risk of Stomach and Other Digestive System Cancers in the Iowa Women’s Health Study
Source: Int J Environ Res Public Health. 2021 Jun 25;18(13):6822. doi: 10.3390/ijerph18136822 (PMC8297261; doi:10.3390/ijerph18136822)
Supplement: Supplementary file 1 [file ijerph-18-06822-s001.zip › ijerph-1231790-supplementary.pdf]

Supplemental Material

Supplemental Table S1: Associations between biliary and stomach cancers and groups with at or above (high) and below median (low) intakes of drinking water nitrate (NO_3-N), vitamin C, daily vegetable intake, daily red meat intake, daily processed meat intake, and smoking status (ever/never).

	Low NO_3-N High Vitamin C[a]	Low NO_3-N Low Vitamin C	High NO_3-N High Vitamin C	High NO_3-N Low Vitamin C	Low NO_3-N High Vegetables[b]	Low NO_3-N Low Vegetables	High NO_3-N High Vegetables	High NO_3-N Low Vegetables
**N**	3,854	3,936	3,860	3,927	3,304	4,486	3,293	4,494
**Biliary tract**								
Cases	12	12	10	16	12	12	6	20
HR[a]	ref	0.9 (0.4-2.0)	0.8 (0.4-1.9)	1.2 (0.6-2.6)	ref	0.7 (0.3-1.6)	0.5 (0.2-1.4)	1.1 (0.5-2.4)
**Stomach**								
Cases	5	11	8	6	7	9	5	9
HR[a]	ref	2.1 (0.7-6.2)	1.6 (0.5-4.9)	1.2 (0.4-3.9)	ref	1.0 (0.3-2.7)	0.7 (0.2-2.3)	1.0 (0.3-2.6)

	Low NO_3-N Low Red Meat[d]	Low NO_3-N High Red Meat	High NO_3-N Low Red Meat	High NO_3-N High Red Meat	Low NO_3-N Low Processed Meat[e]	Low NO_3-N High Processed Meat	High NO_3-N Low Processed Meat	High NO_3-N High Processed Meat
**N**	4,116	3,674	4,247	3,540	3,365	4,425	3,546	4,241
**Biliary tract**								
Cases	12	12	17	9	9	15	8	18
HR[a]	ref	1.2 (0.5-2.9)	1.4 (0.7-2.9)	1.0 (0.4-2.4)	ref	1.4 (0.6-3.1)	0.8 (0.3-2.1)	1.7 (0.8-3.9)
**Stomach**								
Cases	10	6	7	7	6	10	3	11
HR[a]	ref	0.7 (0.2-2.0)	0.7 (0.3-1.8)	0.8 (0.3-2.3)	ref	1.3 (0.5-3.7)	0.5 (0.1-1.9)	1.5 (0.6-4.2)

	Low NO_3-N Never Smoking	Low NO_3-N Ever Smoking	High NO_3-N Never Smoking	High NO_3-N Ever Smoking
**N**	4,882	2,908	4,794	3,540
**Biliary tract**				
Cases	16	8	19	7
HR[a]	ref	1.0 (0.4-2.4)	1.2 (0.6-2.4)	0.9 (0.4-2.1)
**Stomach**				
Cases	12	4	9	5
HR[a]	ref	0.7 (0.2-2.3)	0.8 (0.3-1.8)	0.9 (0.3-2.5)

[a]Adjusted for age, calorie intake, and BMI. [b]Median of vitamin C intake=190.8 mg/day. [c]Median of vegetables intake=200 g/day. [d]Median of red meat intake=79.2 g/day. [e]Median of processed meat intake=1.8 g/day.

Supplemental Table S2. Association between specific digestive system cancers and average drinking water TTHM (μg/L), continuous TTHM (log TTHM), and years above half the maximum contaminant level ([MCL]; N=14,939).[a] Quartile groups for biliary tract cancer. Tertile groups for stomach cancer. Median groups for esophagus, small intestine, gallbladder, bile duct, and liver cancers.

	Average TTHM (μg/L)				Continuous (log TTHM)	Years with >½-MCL (>40 μg/L TTHM)	
**Range**	<0.90	0.91-4.59	4.60-14.31	>14.31		0 years	>0 years
**N**	3,752	3,670	4,139	3,278	15,066	11,265	3,801
**Biliary tract**							
Cases	15	10	18	7	50	43	7
HR[b]	ref	0.7 (0.3-1.6)	1.1 (0.6-2.2)	0.5 (0.2-1.3)	1.0 (0.9-1.1)	ref	0.5 (0.2-1.1)
**Range**	<0.90	0.91-11.5	>11.5	—			
**N**	5,476	5,110	5,324	—			
**Stomach**							
Cases	12	7	10	—	29	25	4
HR[b]	ref	0.7 (0.3-1.9)	0.9 (0.4-2.1)	—	1.0 (0.9-1.1)	ref	0.5 (0.2-1.4)
**Range**	<4.60	>4.60	—	—			
**N**	8,231	7,679	—	—			
**Esophagus**							
Cases	10	9	—	—	19	13	6
HR[c]	ref	0.8 (0.3-2.0)	—	—	1.0 (0.8-1.1)	ref	1.2 (0.5-3.2)
**Small Intestine**							
Cases	10	5	—	—	15	12	3
HR[d]	ref	0.5 (0.2-1.4)	—	—	0.9 (0.8-1.1)	Ref	0.7 (0.2-2.6)
**Gallbladder**							
Cases	15	10	—	—	25	23	2
HR[d]	ref	0.7 (0.3-1.5)	—	—	1.0 (0.9-1.1)	ref	0.3 (0.1-1.1)
**Bile Duct**							
Cases	10	15	—	—	25	20	5
HR[b]	ref	1.5 (0.7-3.4)	—	—	1.0 (0.9-1.2)	ref	0.8 (0.3-2.0)
**Liver**							
Cases	6	4	—	—	10	8	2
HR[e]	ref	0.7 (0.2-2.4)	—	—	1.0 (0.8-1.2)	ref	0.8 (0.2-3.6)

[a]Limited to women 11+ years on PWS, and those with non-missing smoking status. [b]Adjusted for age and BMI. [c]Adjusted for age, smoking status, and alcohol intake. [d]Adjusted for age and farm/rural. [e]Adjusted for age and smoking pack years.
